# Supplementary material for: Domain loss enabled evolution of novel functions in the snake three-finger toxin gene superfamily
Source: Nat Commun. 2023 Aug 11;14:4861. doi: 10.1038/s41467-023-40550-0 (PMC10421932; doi:10.1038/s41467-023-40550-0)
Supplement: Supplementary file 3 — Description of Additional Supplementary Files [file 41467_2023_40550_MOESM3_ESM.pdf]

Description of Additional Supplementary Files

Supplementary Data 1

Description: Dataset in a spreadsheet format, including aligned sequences, grouping and localization predictions, and comparison metrics with existing .pdb structures wehre applicable.

Supplementary Data 2

Description: Protein structures in .pdb format computed for the main dataset in this study.

Supplementary Data 3

Description: TOLIP protein space clustering.

Supplementary Data 4

Description: Protein language models' embeddings.
