# Supplementary material for: Domain loss enabled evolution of novel functions in the snake three-finger toxin gene superfamily
Source: Nat Commun. 2023 Aug 11;14:4861. doi: 10.1038/s41467-023-40550-0 (PMC10421932; doi:10.1038/s41467-023-40550-0)
Supplement: Supplementary file 8 — Reporting Summary [file 41467_2023_40550_MOESM8_ESM.pdf]

nature portfolio

Corresponding author(s): Koludarov Ivan

Last updated by author(s): Jun 22, 2023

Reporting Summary

Nature Portfolio wishes to improve the reproducibility of the work that we publish. This form provides structure for consistency and transparency in reporting. For further information on Nature Portfolio policies, see our Editorial Policies and the Editorial Policy Checklist.

Statistics

For all statistical analyses, confirm that the following items are present in the figure legend, table legend, main text, or Methods section.

n/a	Confirmed	
☒	☐	The exact sample size (*n*) for each experimental group/condition, given as a discrete number and unit of measurement
☒	☐	A statement on whether measurements were taken from distinct samples or whether the same sample was measured repeatedly
☐	☒	The statistical test(s) used AND whether they are one- or two-sided<br>*Only common tests should be described solely by name; describe more complex techniques in the Methods section.*
☒	☐	A description of all covariates tested
☒	☐	A description of any assumptions or corrections, such as tests of normality and adjustment for multiple comparisons
☒	☐	A full description of the statistical parameters including central tendency (e.g. means) or other basic estimates (e.g. regression coefficient) AND variation (e.g. standard deviation) or associated estimates of uncertainty (e.g. confidence intervals)
☐	☒	For null hypothesis testing, the test statistic (e.g. *F*, *t*, *r*) with confidence intervals, effect sizes, degrees of freedom and *P* value noted<br>*Give P values as exact values whenever suitable.*
☐	☒	For Bayesian analysis, information on the choice of priors and Markov chain Monte Carlo settings
☒	☐	For hierarchical and complex designs, identification of the appropriate level for tests and full reporting of outcomes
☒	☐	Estimates of effect sizes (e.g. Cohen's *d*, Pearson's *r*), indicating how they were calculated

Our web collection on statistics for biologists contains articles on many of the points above.

Software and code

Policy information about availability of computer code

Data collection	Not applicable, because all data used are from publicly available databases
Data analysis	NCBI-BLAST v2.7.1+, Geneious Prime 2022.2.1, MAFFT v7.305, ExaBayes v1.5.1, iqTree v1.6.1, bio_embeddings v0.2.2, RostSpace v0.1.1, ColabFold v1.3.0, oldseek v6.29e2557, R v3.6.2, RStudio v2023.03.0, DALI v5, TMbed v1.0.0, UMAP v0.5.3, DBSCAN v1.1-10, CLANS v29.05.2012

For manuscripts utilizing custom algorithms or software that are central to the research but not yet described in published literature, software must be made available to editors and reviewers. We strongly encourage code deposition in a community repository (e.g. GitHub). See the Nature Portfolio guidelines for submitting code & software for further information.

Data

Policy information about availability of data

All manuscripts must include a data availability statement. This statement should provide the following information, where applicable:

- Accession codes, unique identifiers, or web links for publicly available datasets
- A description of any restrictions on data availability
- For clinical datasets or third party data, please ensure that the statement adheres to our policy

All data available on Zenodo: DOI https://doi.org/10.5281/zenodo.8069952

Research involving human participants, their data, or biological material

Policy information about studies with human participants or human data. See also policy information about sex, gender (identity/presentation), and sexual orientation and race, ethnicity and racism.

Reporting on sex and gender	N/A
Reporting on race, ethnicity, or other socially relevant groupings	N/A
Population characteristics	N/A
Recruitment	N/A
Ethics oversight	N/A

Note that full information on the approval of the study protocol must also be provided in the manuscript.

Field-specific reporting

Please select the one below that is the best fit for your research. If you are not sure, read the appropriate sections before making your selection.

[x] Life sciences [ ] Behavioural & social sciences [ ] Ecological, evolutionary & environmental sciences

For a reference copy of the document with all sections, see nature.com/documents/nr-reporting-summary-flat.pdf

Life sciences study design

All studies must disclose on these points even when the disclosure is negative.

Sample size	In our computational study, the "sample size" comprises the genomes of 15 representative tetrapodan species, 1,427 protein sequences (3FTx) as the main dataset, and 2,466 sequences from the TOLIP family as a supplementary dataset. These sequences and genomes were chosen as they represent the complete collection of relevant data currently available and provide a comprehensive overview of all major tetrapodan clades. This exhaustive approach, inherent to our computational design, ensures the widest coverage and maximises the generalisability of our results.
Data exclusions	The only data excluded from the analysis were fragmented sequences. This exclusion criterion was pre-established to maintain data integrity and ensure reliable results, as incomplete sequences could potentially introduce bias or inaccuracies into our computational models and subsequent analysis.
Replication	The notion of replication in the traditional experimental sense is not applicable to our computational study. However, the analyses were conducted consistently using the same computational methods across the entire dataset, which can be considered analogous to replication. Our methodologies are also transparent and repeatable by others, ensuring the robustness and reproducibility of our findings.
Randomization	Randomisation, often used to prevent selection bias in experimental studies, is not directly applicable to our computational study. Given the comprehensive nature of our dataset, which includes all usable sequences, no selection bias could be introduced. The analyses performed did not benefit from randomisation, as the entirety of the available data was systematically and uniformly processed.
Blinding	The concept of blinding, typically applied to reduce bias in experimental studies, is not relevant to our computational study. Our computational analyses were executed in an unbiased manner, following predetermined methods and protocols, irrespective of the data. There are no subjective decisions or observations in this computational process that could introduce bias, making the procedure inherently "blind".

Reporting for specific materials, systems and methods

We require information from authors about some types of materials, experimental systems and methods used in many studies. Here, indicate whether each material, system or method listed is relevant to your study. If you are not sure if a list item applies to your research, read the appropriate section before selecting a response.

Materials & experimental systems

n/a	Involved in the study
☒	☐ Antibodies
☒	☐ Eukaryotic cell lines
☒	☐ Palaeontology and archaeology
☒	☐ Animals and other organisms
☒	☐ Clinical data
☒	☐ Dual use research of concern
☒	☐ Plants

Methods

n/a	Involved in the study
☒	☐ ChIP-seq
☒	☐ Flow cytometry
☒	☐ MRI-based neuroimaging
