# Supplementary material for: Domain loss enabled evolution of novel functions in the snake three-finger toxin gene superfamily
Source: Nat Commun. 2023 Aug 11;14:4861. doi: 10.1038/s41467-023-40550-0 (PMC10421932; doi:10.1038/s41467-023-40550-0)
Supplement: Supplementary file 1 — Supplementary Information [file 41467_2023_40550_MOESM1_ESM.pdf]

Supplementary Figures to Domain loss enabled evolution of novel functions in the snake three-finger toxin gene superfamily

Ivan Koludarov[1,*,^], Tobias Senoner[1,2*], Timothy N. W. Jackson[3], Daniel Dashevsky[4], Michael Heinzinger[1], Steven D. Aird[5] & Burkhard Rost[1, 6]

1 TUM (Technical University of Munich) Department of Informatics, Bioinformatics & Computational Biology - i12, Boltzmannstr. 3, 85748 Garching/Munich, Germany
2 TUM Graduate School, Center of Doctoral Studies in Informatics and its Applications (CeDoSIA), Boltzmannstr. 11, 85748 Garching/Munich, Germany
3 Australian Venom Research Unit, Department of Biochemistry and Pharmacology, University of Melbourne, Australia
4 Australian National Insect Collection, Commonwealth Scientific & Industrial Research Organisation, Canberra, Australia
5 7744-23 Hotaka Ariake, Azumino-shi, Nagano-ken, Japan 399-8301
6 Institute for Advanced Study (TUM-IAS), Lichtenbergstr. 2a, 85748 Garching/Munich, Germany & TUM School of Life Sciences Weihenstephan (WZW), Alte Akademie 8, Freising, Germany
* Contributed equally
^ Corresponding author: koludarov@rostlab.org, www.rostlab.org, +49-289-17-811 (email rost: assistant@rostlab.org)

1. ***TOP1MT* region gene map figure**
2. **Phylogenetic trees**
    a) DALI
    b) iqTree
    c) Exabayes
3. **CLANS analysis**
4. **DBSCAN clusters**

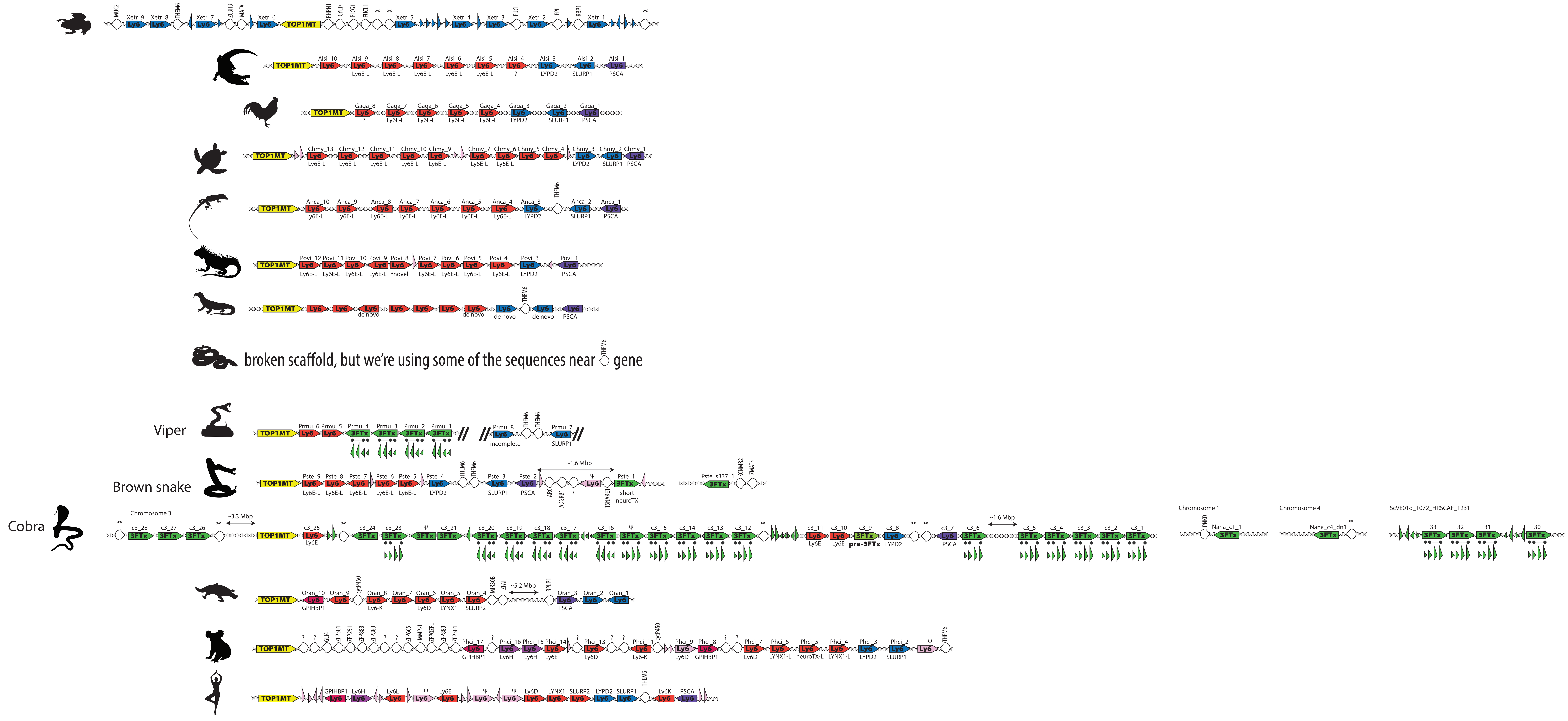

Supplementary Figure 1: Gene maps for *TOP1MT* genomic region of representative species. Yellow arrow marks *TOP1MT* gene, other colored arrows mark *Ly6* genes colored by orthology, white pointed semicircles mark any other gene present in the cluser (names given above). Triangles in line with the genes repre- sent orphan *Ly6* exons colored by inferred homology. Triangles under *3FTx* genes mark exonic structure of those genes that have non-canonical 4 exon structure, with a 3rd exon always pseudogenised. Names under the genes are given exactly as they are in genomic annotations. Names on top of the genes stand for the code used in our analysis.

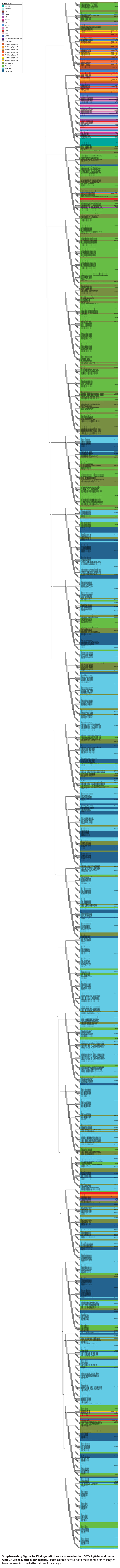

Supplementary Figure 2a: Phylogenetic tree for non-redundant 3FTx/Ly6 dataset made with DALI (see Methods for details). Clades colored according to the legend, branch lengths have no meaning due to the nature of the analysis.

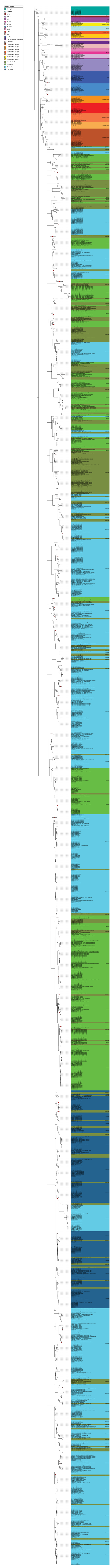

Supplementary Figure 2b: Phylogenetic tree for non-redundant 3FTx/Ly6 dataset made with iqTRee(see Methods for details). Clades colored according to the legend, nodes with boostrap value higher than 85 are marked with a red dot.

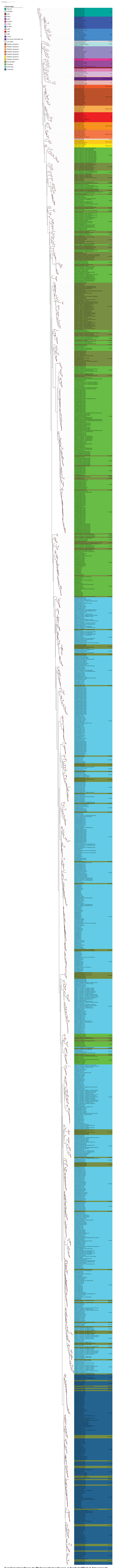

Supplementary Figure 2c: Phylogenetic tree for non-redundant 3FTx/Ly6 dataset made with ExaBayes (see Methods for details). Clades colored according to the legend, nodes with posterior probability value higher than 85 are marked with a red dot.

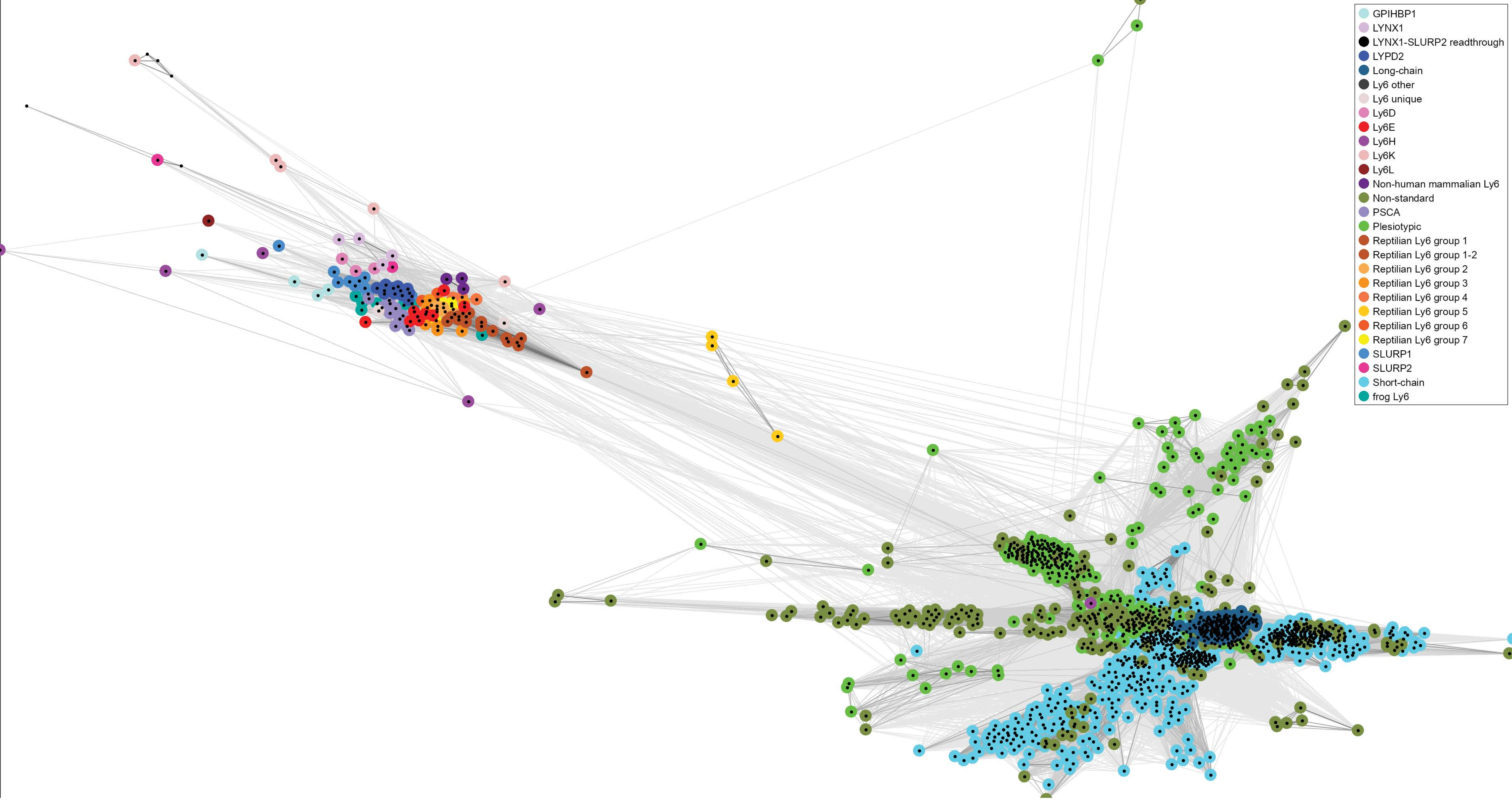

Supplementary Figure 3: CLANS sequence similarity based clustering for the non-redundant 3FTx/Ly6 dataset. Datapoints colored according to the legend. The process involved an all-against-all BLAST comparison via the MPI Bioinformatics Toolkit's online CLANS tool. The image was produced using the CLANS Java application in 2D mode with 10,000 iterations, employing a p-value cutoff of > 1e-5. Please note that CLANS utilizes pairwise BLAST comparisons, not traditional statistical tests, thus concepts like one-sided or two-sided testing or adjustments for multiple comparisons are not applicable. BLAST HSP E-value was set to the 1e-4 (default). The P-value cutoff of > 1e-5 was used in the CLANS analysis to display connection lines.

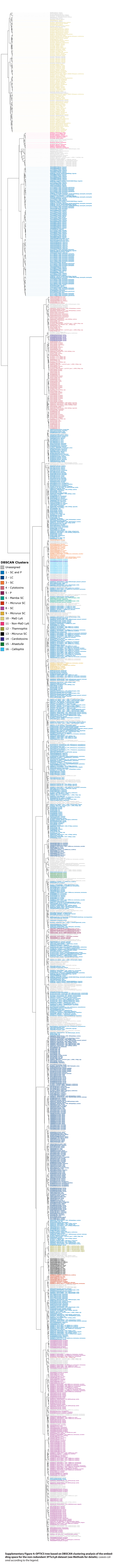

Supplementary Figure 4: OPTICS tree based on DBSCAN clustering analysis of the embedding space for the non-redundant 3FTx/Ly6 dataset (see Methods for details). Leaves colored according to the legend.
